# Absolute requirement of cholesterol binding for Hedgehog gradient formation in *Drosophila*

**DOI:** 10.1242/bio.20134952

**Published:** 2013-05-09

**Authors:** Antoine Ducuing, Bertrand Mollereau, Jeffrey D. Axelrod, Stephane Vincent

**Affiliations:** 1 LBMC, UMR5239 CNRS/Ecole Normale Supérieure de Lyon, SFR 128 Biosciences Lyon Gerland, Université de Lyon, 69364 Lyon Cedex 07, France; 2 Pathology Department, Stanford School of Medicine, Palo Alto, CA 94304, USA

**Keywords:** *Drosophila*, Hedgehog, Cholesterol, Gradient, Patterning

## Abstract

How morphogen gradients are shaped is a major question in developmental biology, but remains poorly understood. Hedgehog (Hh) is a locally secreted ligand that reaches cells at a distance and acts as a morphogen to pattern the *Drosophila* wing and the vertebrate neural tube. The proper patterning of both structures relies on the precise control over the slope of Hh activity gradient. A number of hypotheses have been proposed to explain Hh movement and hence graded activity of Hh. A crux to all these models is that the covalent binding of cholesterol to Hh N-terminus is essential to achieve the correct slope of the activity gradient. Still, the behavior of cholesterol-free Hh (Hh-N) remains controversial: cholesterol has been shown to either increase or restrict Hh range depending on the experimental setting. Here, in fly embryos and wing imaginal discs, we show that cholesterol-free Hh diffuses at a long-range. This unrestricted diffusion of cholesterol-free Hh leads to an absence of gradient while Hh signaling strength remains uncompromised. These data support a model where cholesterol addition restricts Hh diffusion and can transform a leveled signaling activity into a gradient. In addition, our data indicate that the receptor Patched is not able to sequester cholesterol-free Hh. We propose that a morphogen gradient does not necessarily stem from the active transfer of a poorly diffusing molecule, but can be achieved by the restriction of a highly diffusible ligand.

## Introduction

The Hedgehog (Hh) gene family encodes secreted ligands that regulate patterning in both vertebrates and invertebrates (Ingham and McMahon, 2001; Ingham et al., 2011). The range of action of Hh ligands determines patterns of prominent body structures such as the segments in the fly embryo, the appendages in both the adult fly and vertebrates (Riddle et al., 1993; Tabata and Kornberg, 1994) and the ventral neural tube in vertebrates (Jessell, 2000). Hh regulates its targets in a concentration-dependent manner, and thus acts as a morphogen in the *Drosophila* wing imaginal disc and the vertebrate neural tube: Hh is secreted locally and its range of action patterns distinct territories (Briscoe et al., 2001; McMahon et al., 2003). Hh differential activity must therefore be carefully controlled.

Two opposing views may explain how the slope of a morphogen gradient is generated: First, a freely diffusible molecule can encounter a restrictive mechanism, leading to its accumulation near the source of secretion. Up to now, such hypothesis has received little support. Second, a poorly diffusible molecule could be transferred upon interaction with a carrier in order to reach the cells that need to be patterned. Distinct transfer mechanisms have been proposed to explain gradient formation in the *Drosophila* wing imaginal disc (Kornberg and Guha, 2007): First, during serial transfer also known as trancytosis, secreted Hh would be endocytosed by the neighboring cell in a receptor-dependent manner, and then secreted again. Repeating this scenario in the rest of the cells in the epithelium will lead to the formation of the gradient. Second, lipoprotein particle transfer would involve the binding of Hh to lipophorin. The Hh–lipophorin complex would move across the tissue, allowing long-range signaling (Panáková et al., 2005; Eugster et al., 2007). Third, Hh may be transferred by long cellular protrusions called cytonemes (Ramírez-Weber and Kornberg, 1999). Cells interpreting a ligand would send specific cytonemes bearing a receptor to pick up the ligand at the secretion site (Roy et al., 2011). Another possibility is that the cytonemes originate from the Hh producing cells as shown in the niche of the *Drosophila* female germline stem cells (Rojas-Ríos et al., 2012). Recently, cytonemes have also been shown to originate from the Hh producing cells in the wing imaginal disc (Bilioni et al., 2013). The question of how Hh activity gradient is established is therefore highly controversial and remains open. The underlying idea behind these models is that a transfer mechanism carries local Hh in order to generate an activity gradient with a precise slope.

Hh protein biosynthesis includes the addition of palmitic acid and cholesterol to the N moiety (Hh-N) (reviewed by Mann and Beachy, 2004). Hh is palmitoylated at its N-terminus by the acetyl transferase *skinny hedgehog* and is required for Hh secretion (Chamoun et al., 2001; Micchelli et al., 2002). The second lipid modification is the covalent addition of a cholesterol moiety. Cholesterol addition requires the autocatalytic Hh C-terminal domain that gets cleaved during the reaction (Porter et al., 1996a; Bürglin, 2008). Cholesterol covalent binding is crucial for Hh release mediated by the transmembrane protein Dispatched (Disp) that contains a sterol-sensing domain (Burke et al., 1999). Still, expressing the Hh N-terminal domain alone produces a form of Hh not bound to cholesterol that is efficiently secreted in a *disp* independent manner (Porter et al., 1996b; Burke et al., 1999). Hh-N was used to show that cholesterol addition enhances membrane association (Porter et al., 1996b). The more striking behavior of Hh-N is its range of action that is different from the one of the wild-type, cholesterol bound form of Hh. The problem is that depending on experimental conditions, the cholesterol adduct would increase (Gallet et al., 2003; Panáková et al., 2005; Gallet et al., 2006; Eugster et al., 2007) or decrease (Porter et al., 1996b; Burke et al., 1999; Dawber et al., 2005; Callejo et al., 2006; Su et al., 2007) Hh range of action (reviewed by Wendler et al., 2006).

It was first found that cholesterol addition limits Hh diffusion, as predicted from its biochemical properties (Porter et al., 1996b; Burke et al., 1999). In wing imaginal discs, Hh-N would diffuse further than the wild-type tending to decrease the slope of its gradient and thus reducing peak levels while elevating low levels at a distance. In this case, the domains of the high-threshold targets *patched (ptc)* and *engrailed (en)* would decrease in size or may even get lost (Dawber et al., 2005; Callejo et al., 2006; Gallet et al., 2006). On the other hand, Hh-N can activate the low-threshold targets *Collier* and *Iroquoi* over a greater range than Hh-WT (Dawber et al., 2005; Callejo et al., 2006). Besides, the direct analysis of the spreading of Hh GFP fusions showed that the Hh-N-GFP would diffuse twice further than Hh-GFP (Su et al., 2007). Therefore this model suggests that the cholesterol moiety concentrates Hh in a given domain above the activation threshold of the pathway and defines the effective range of Hh (Guerrero and Chiang, 2007).

Still, other data indicated that cholesterol binding could be used to increase Hh range of action: wing imaginal disc clones overexpressing Hh-N induced the expression of the target reporter *dpp-lacZ* at a range of 3 to 4 cells whereas similar clones overexpressing Hh-WT induce *dpp-lacZ* at a range of 5 to 6 cells (Gallet et al., 2006). In the embryo, whereas it was first shown that Hh-N diffuses more than Hh-WT (Burke et al., 1999), it was later proposed that cholesterol binding is necessary for Hh movement (Gallet et al., 2003; Gallet et al., 2006). The hydrophobic nature of cholesterol and the longer range observed were reconciled by the observation that the cholesterol adduct promotes the association of Hh into lipoparticles able to travel in the extracellular matrix (Greco et al., 2001; Panáková et al., 2005; Eugster et al., 2007). Indeed, Hh copurifies with lipophorin, and Hh range of action decreases when lipophorin levels were reduced with RNAi in *Drosophila* larvae. As a result, *dpp-lacZ* expression decreased from 11 to 6 rows of cells at the anteroposterior boundary of wing imaginal disc (Panáková et al., 2005). Hence, the cholesterol adduct appeared to increase Hh range by a factor of 2. Inexplicably, the expression range of the other Hh target *Collier (Col)* was unaffected. Another difficulty with this model is that lipoparticles are known to carry GPI-anchored proteins, but GPI-anchored Hh does not diffuse (Burke et al., 1999). Cholesterol binding would therefore provide a way by which a poorly diffusing molecule could get transferred to the neighboring cells.

Altogether the control of Hh range of action by cholesterol modification is unclear: in the *Drosophila* embryo it is admitted that cholesterol modification increases Hh range of action. In discs, Hh-N range of action was either described as decreasing by a factor of 2 (Gallet et al., 2003; Gallet et al., 2006) or increasing by a factor of 2 although only for low-threshold targets (Dawber et al., 2005; Callejo et al., 2006; Su et al., 2007). Most of all, the wider implication of these studies is that cholesterol binding does not change Hh behavior in a drastic manner, but only tunes the shape of the gradient. The process of cholesterol binding would therefore be dispensable to the formation of the gradient itself.

Our data in both the *Drosophila* embryo and the wing imaginal disc show a dramatic increase in the range of Hh-N. Cholesterol-bound or unbound Hh was expressed in the embryonic dorsal epidermis and the activity of Hh pathway was monitored along an axis perpendicular to the direction of endogenous Hh diffusion. This setting allowed us to demonstrate that Hh-N can act at a long range in the *Drosophila* embryo, as far as 25 cells away. Second, we show that cholesterol-free Hh displays unrestricted diffusion in the wing disc by using *ptc* expression as a readout. This unrestricted diffusion leads to an absence of activity gradient. This plateau of Hh activity is still able to induce high threshold targets such as En, indicating that Hh-N is potent enough to induce full Hh pathway activation, implying that the longer range is not obtained at the expanse of the strength of the signal. We conclude that cholesterol modification is essential for Hh gradient formation.

## Materials and Methods

### Fly strains and genetics

We used the *hh^ts2^* (# BL 1684), a temperature sensitive allele with restrictive temperature at 29°C. To drive ectopic expression with the UAS/Gal4 system (Brand et al., 1994), we used the following Gal4 lines: *pnr-Gal4* (*pnr^MD237^*, # BL 3039) which drives expression in the dorsal epidermis of the embryo, and *ap-Gal4* (*ap^MD544^*, # BL 3041) which drives expression in the dorsal domain of the wing disc. We used the following UAS lines: *UAS-ActinRFP, UAS-hh-WT* (Gallet et al., 2003), *UAS-hh-N* (Gallet et al., 2003), *UAS-hh::GPI*, a fusion of FasI C-terminal residues that include a GPI anchoring signal with the Hh-N moiety (Burke et al., 1999) and *UAS-Hh::CD2*, a fusion of the rat membrane protein CD2 with the Hh-N moiety (Strigini and Cohen, 1997). We also used the *Dpp-lacZ* line BS3.0 (Blackman et al., 1991). *pnr^MD237^*, *ap^MD544^*, *UAS-RFP* and *hh^ts2^* lines are from the Bloomington *Drosophila* stock centre. *UAS-hh-WT*, *UAS-hh-N*, *UAS-hh::GPI*, *UAS-hh::CD2* are a kind gift from Armel Gallet. The *Dpp-lacZ* reporter is a kind gift from L.S. Shashidhara. Crosses were performed at 25°C. For the *hh^ts2^* experiment, larvae were incubated at restrictive temperature (29°C) 19 hours before dissection.

### Immunofluorescence and quantification

We used standard techniques of immunohistofluorescence: embryos were dechorionated with bleach, fixed in a 1:1 mix of 4% PFA–Heptane. Embryos were subsequently devitellinized by replacing the 4% PFA with methanol. Discs were fixed in 4% PFA on ice for 1 hour. Samples were then incubated with primary antibodies, later fluorescent-coupled secondary antibodies. Samples were eventually mounted in VectaShield. We used the following primary antibodies: anti-Odd (kind gift from J. Skeath), anti-Ci, anti-En, anti-Ptc, anti-DCadherin, anti-Wg, developed respectively by R. Holmgren, C. Goodman, I. Guerrero, T. Uemura, S. Cohen, were obtained from the Developmental Studies Hybridoma Bank developed under the auspices of the NICHD and maintained by the University of Iowa, Department of Biology, Iowa City, IA 52242. Anti-β-Gal is from Cappel. We used the following secondary antibodies: Alexa Donkey anti-Mouse 488 (Invitrogen), Alexa Goat anti-Mouse 633 (Invitrogen), Alexa Goat anti-Rat 633 (Invitrogen), Alexa anti-Rabbit 633 (Invitrogen). Images were acquired on the Confocal Leica SP5 microscope and analysed with ImageJ. Unless otherwise indicated, all images are projections of confocal sections. For all panels, scale bar is 10 µm. ImageJ plot profile function was used to quantify Ptc intensity for Figs 4 and 5.

### Western blot

We used the same protocol as previously described (Dourlen et al., 2012). 100 embryos or 20 wing imaginal discs for each genotype were homogenized in Laemmli buffer (10% glycerol, pH 6.8 0.5M Tris, 10% SDS, 1% bromophenol blue, 1% β-mercaptoethanol, 100 mM DTT). Samples were then boiled and loaded onto a 12% acrylamide gel (Biorad), transferred and incubated overnight at 4°C with a primary antibody. Samples were then incubated with HRP-coupled secondary antibodies, and eventually detected with a chemoluminescent kit (GE Healthcare Life Sciences). The following antibodies were used: “Calvados” Anti-Hh (kind gift from P. Thérond) and Anti-Tubulin (Sigma). We used the following secondary antibodies: Anti-mouse HRP and Anti-rabbit HRP antibodies (Biorad). We used the ImageJ software to quantify protein bands.

### Statistical analyses

**Fig. 1.**
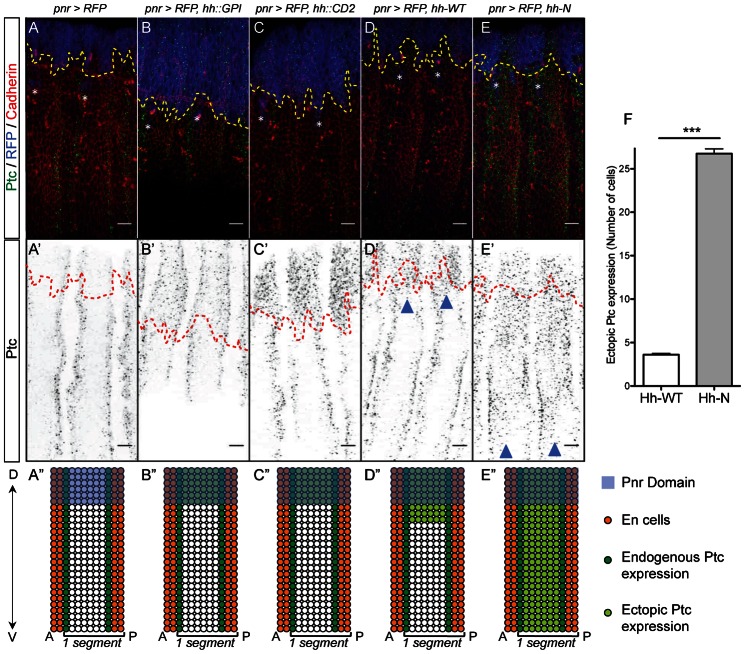
Hh-N activates Ptc expression ten times further than Hh-WT in the *Drosophila* embryo. (**A–E′**) Ptc, Cadherin and RFP expression in stage 13 embryos. The ectopic expression domain is located above the dashed lines. Asterisks indicate underlying Pnr-positive PNS neurons. (A–C′) Control embryos. Endogenous Ptc is detected in 1-cell wide stripes abutting the En domain (A,A′). Both Hh:GPI, and Hh::CD2 induce Ptc cell-autonomously (B′–C′). (D–E′) In *pnr-Gal4, UAS-RFP, UAS-hh-WT* embryos, Ptc is induced at a 3-cell range inside the lateral epidermis whereas in *pnr-Gal4, UAS-RFP, UAS-hh-N* embryos, Ptc is induced throughout the epidermis (D–E′, arrowheads). (**A″–E″**) Schematics representing segments of the above genotypes. Ectopic Ptc is in light green. (**F**) Quantification of ectopic Ptc expression range (*n≥8*, *P-value = 0.0003*). Scale bars: 10 µm.

**Fig. 2.**
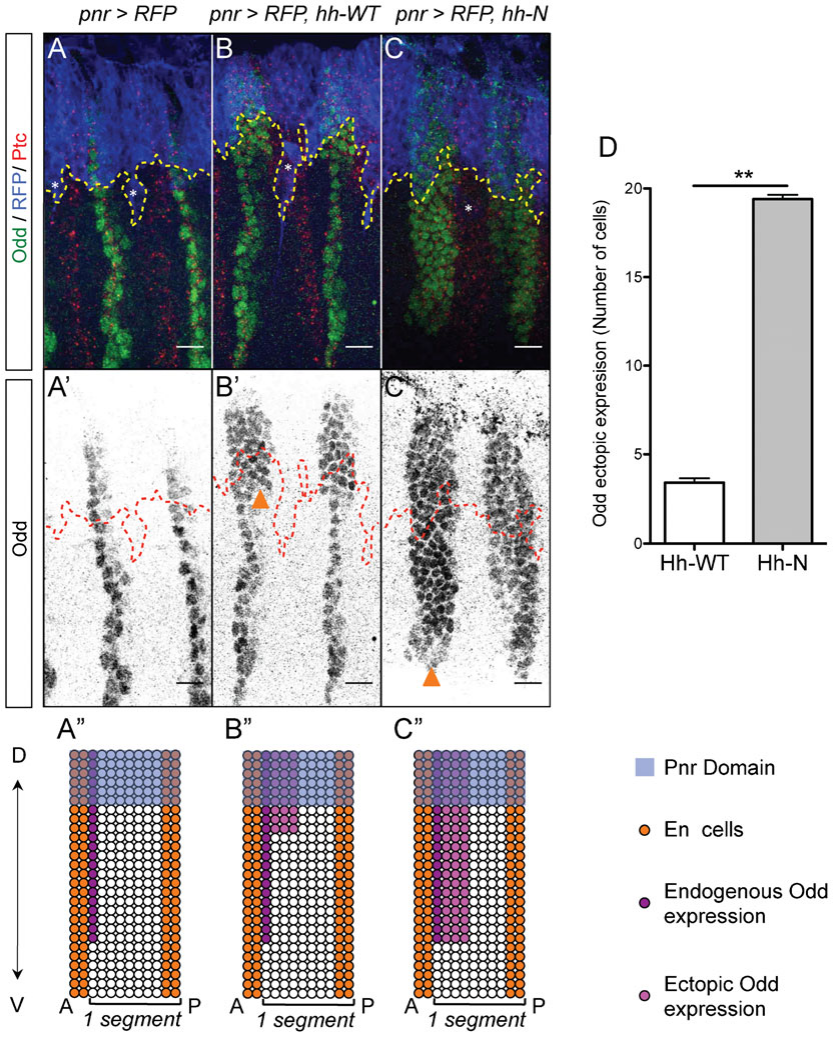
Hh-N maintains Odd expression at a long-range in the *Drosophila* embryo. (**A–C′**) Odd, Ptc and RFP expression in stage 13 embryos. The ectopic expression domain is located above the dashed lines. Asterisks indicate underlying Pnr-positive PNS neurons. (A,A′) Endogenous Odd is detected in a 1-cell wide stripe in the dorsal and the lateral epidermis. (B,B′) Hh-WT maintains Odd only 3 cells away from the pnr domain, whereas Hh-N maintains a 4-cell wide stripe of Odd cells all through the lateral epidermis. (**A″–C″**) Schematics representing segments of the above genotypes. Ectopic Odd is in magenta. (**D**) Quantification of ectopic Odd expression range (*n≥5*, *P-value = 0.0097*). Scale bars: 10 µm.

We used the Prism software to generate graphs. Bar graphs represent mean±s.e.m. Mann–Whitney's *U* test was used to determine significant differences for Figs 1, 2. Student *t*-test was used to determine significant differences for Fig. 4.

## Results

### Unrestricted diffusion of cholesterol-free Hh in the *Drosophila* embryo

*hh* is a segment polarity gene (Nüsslein-Volhard and Wieschaus, 1980) that regulates patterning within each segment of the *Drosophila* embryo. Hh is secreted by the *en*-expressing cells (Kornberg et al., 1985) and induces *ptc* expression in the *Ci*-expressing domain. Ptc expression is detected in all Ci positive cells at early stage 10 (Taylor et al., 1993) and is refined to single stripes of cells abutting the En domain at stage 13 (Fig. 1A–A″). We therefore characterized the range achieved by different Hh variants by monitoring ectopic Ptc expression in stage 13 embryos. We used the *pannier-Gal4* (*pnr-Gal4*) driver to overexpress Hh variants in the dorsal domain, marked with Actin-RFP (Calleja et al., 1996) (supplementary material Fig. S1A). Whereas previous experiments had tested Hh range of action across few cell diameters, this setup enabled us to test the range of Hh over 25 cells.

We first overexpressed Hh::GPI and Hh::CD2, two membrane-anchored forms of Hh (Strigini and Cohen, 1997; Burke et al., 1999) as controls and showed that they induce Ptc only within the Pnr domain (Fig. 1B–C″). We next overexpressed cholesterol-bound and cholesterol-free Hh. Ptc staining indicated that wild-type Hh diffuses 1 to 4 cells away (Fig. 1D–D″) whereas cholesterol-free Hh (Hh-N) diffuses throughout the dorsoventral axis (Fig. 1E–E″), which is about 25 cells away (Fig. 1F). Western blot analysis indicates that the greater range of Hh-N is not due to a stronger expression of the Hh-N transgene (supplementary material Fig. S2A,B). Therefore, without cholesterol, Hh diffuses much further than wild-type Hh.

Next, we verified that the activation of the Hh pathway is sufficient to regulate cell identity. In cells posterior to the En cells, *hh* maintains *odd skipped* (*odd*) expression and segmental groove identity (Vincent et al., 2008). In *pnr-Gal4, UAS-RFP* embryos, endogenous Odd expression is wild-type and consists of a single stripe of cells abutting the En domain (Fig. 2A–A″). Hh-WT maintains Odd to about 3 to 4 cells away, correlating perfectly with Ptc expression (Fig. 2B–B″; supplementary material Fig. S3). By contrast, Hh-N maintains Odd throughout the dorsolateral axis (Fig. 2C–C″; supplementary material Fig. S3), which is about 20 cells away (Fig. 2D). This correlation between Ptc expression and Odd maintenance shows that the dose of Hh received by distant cells is strong enough to modify segmental patterning. At this stage, *odd* is not expressed in the ventral epidermis of wild-type embryos (Vincent et al., 2008) and cannot indicate whether Hh-N is active in this region. In order to address whether Hh-N diffuses all the way to the ventral epidermis, we next monitored the pattern of *wg*-expressing cells.

**Fig. 3.**
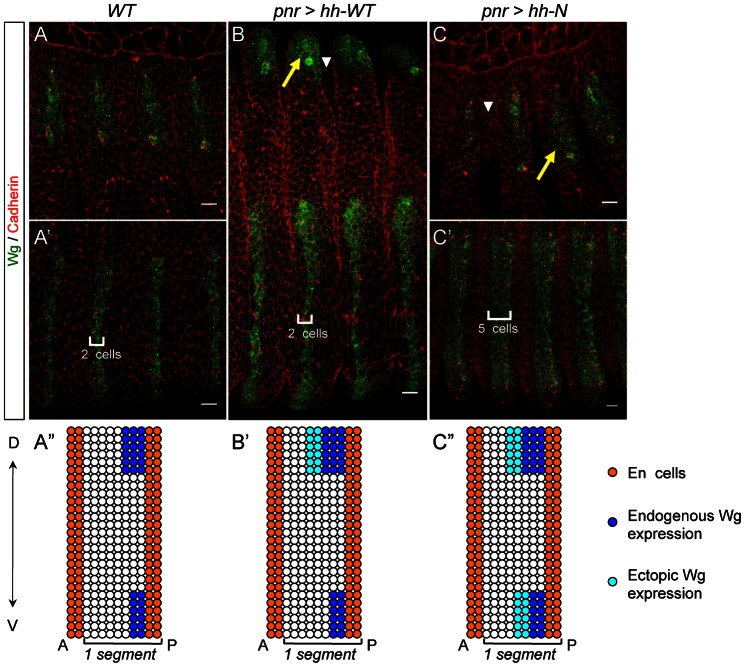
Hh-N maintains Wg expression at a long-range in the *Drosophila* embryo. (**A,A′,B,C,C′**) Stage 13 embryos stained for Wg and Cadherin. (A,A′) *WT* embryos. Wg is expressed in anterior cells of the dorsal epidermis, and in a 2-cell wide stripe in the ventral epidermis. (B,C,C′) Embryos overexpressing Hh-WT or Hh-N exhibit a wider Wg domain (arrows) and wider grooves (arrowheads) in the dorsal epidermis. Only embryos overexpressing Hh-N exhibit ectopic Wg in the ventral epidermis. (**A″,B′,C″**) Schematics representing a segment of the above genotypes. Ectopic Wg is in cyan. Scale bars: 10 µm.

In the dorsal and the ventral epidermis of the embryo, *hh* maintains *wg* expression in cells anterior to the En stripe (Baker, 1987; Alexandre et al., 1999) (Fig. 3A–A″). In *pnr-Gal4, UAS-hh-WT* embryos, supernumerary Wg-expressing cells are detected in the dorsal epidermis but not in the ventral epidermis (Fig. 3B,B′). By contrast, in *pnr-Gal4, UAS-hh-N* embryos, additional rows of Wg-expressing cells are maintained in both the dorsal epidermis and the ventral epidermis (Fig. 3C–C″). Thus Hh-N produced in the dorsal domain diffuses as far as the midline of the ventral epidermis, about 25 cells away. Hence, we conclude that cholesterol-free Hh can diffuse and modify patterning at least ten times further than cholesterol-bound Hh.

### Unrestricted diffusion and absence of gradient with cholesterol-free Hh in the wing imaginal disc

**Fig. 4.**
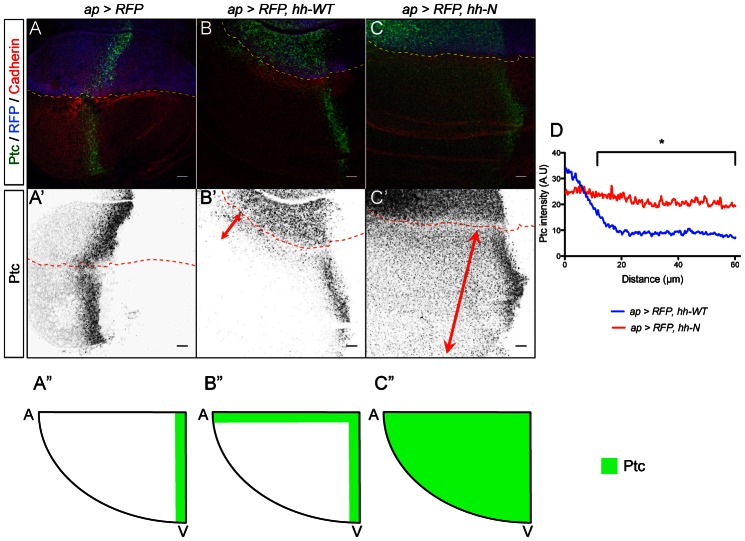
Hh-N induces a long-range plateau of Ptc expression in the *Drosophila* wing disc. (**A–C′**) Ptc, Cadherin and RFP expression in wing imaginal discs. The expression domain is located above the dashed lines. (A,A′) Control discs: a 10-cell stripe abutting the A/P border expresses Ptc. (B–C′) In *ap-Gal4, UAS-RFP, UAS-hh-WT* discs, ectopic Ptc is detected at a 10-cell range whereas in *ap-Gal4, UAS-RFP, UAS-hh-N* Ptc expression expends all throughout the anteroventral quadrant (arrows). (**A″–C″**) Schematics representing anteroventral quadrants of the above genotypes. Ectopic Ptc is in green. (**D**) Quantification of ectopic Ptc expression revealing Hh-WT activity gradient and Hh-N longrange plateau (*n≥6*, for distances >12 µm *P-value<0.05*). Scale bars: 10 µm.

**Fig. 5.**
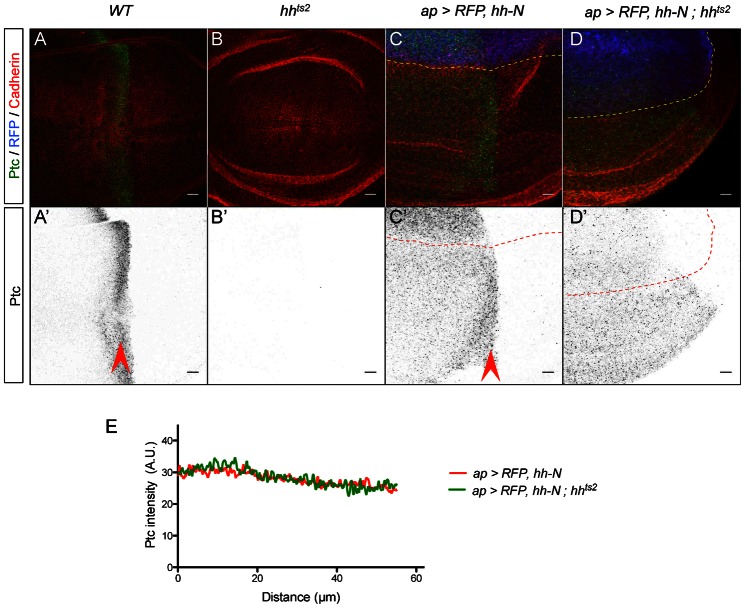
The plateau of Ptc expression induced by Hh-N is independent from endogenous Hh. (**A–D′**) Ptc, Cadherin and RFP expression in wing imaginal discs raised for 19 hours at restrictive temperature (29°C). (A–B′) Control discs. The endogenous Ptc stripe is visible in WT discs (arrowheads) and absent in *hh^ts2^* discs. (C–D′) In both *ap-Gal4, UAS-RFP, UAS-hh-N* and *ap-Gal4, UAS-RFP, UAS-hh-N ; hh^ts2^* discs, ectopic Ptc is detected throughout the anteroventral quadrant. (**E**) Quantification of ectopic Ptc expression revealing homogenous Hh-N activity (*n≥4*). Scale bars: 10 µm.

We next adopted a similar strategy in the wing imaginal disc and tested Hh-N range of action. In the wing imaginal disc, Hh is produced by the posterior *en* cells and activates Ptc in a 10-cell stripe bordering the *en* domain (Fig. 4A–A″). In order to avoid the influence of endogenous Hh activity, we ectopically expressed Hh variants in the dorsal domain with *ap-Gal4* and analyzed their range of action in the anteroventral domain (Calleja et al., 1996; Glise et al., 2005; Ranieri et al., 2012) (supplementary material Fig. S1B). In *ap-Gal4, UAS-RFP, UAS-hh-WT* discs, ectopic Ptc is detected in a stripe of 10 cells along the dorsoventral border (Fig. 4B–B″). In *ap-Gal4, UAS-RFP, UAS-hh-N* discs, ectopic Ptc is detected throughout the anteroventral quadrant of the wing pouch (Fig. 4C–C″). Thus, cholesterol-free Hh induces Ptc expression at least ten times further than cholesterol-bound Hh. Western blot analysis indicates that Hh-N greater range is not due to a stronger expression of the Hh-N transgene (supplementary material Fig. S2C,D). To verify that endogenous Hh does not interfere with these results, we overexpressed Hh-N in a *hh^ts2^* background raised at restrictive temperature during the 19 hours preceding dissection. We observed a similar broad Ptc ectopic expression and an absence of the endogenous Ptc expression (Fig. 5A–D′). Quantitative analysis of Ptc expression reveals that no gradient forms in response to Hh-N (Fig. 4D, Fig. 5E). This is striking as Ptc is a high-threshold Hh target and was strictly detected in a cell-autonomous manner during clonal ectopic expression of Hh-N (Callejo et al., 2006).

**Fig. 6.**
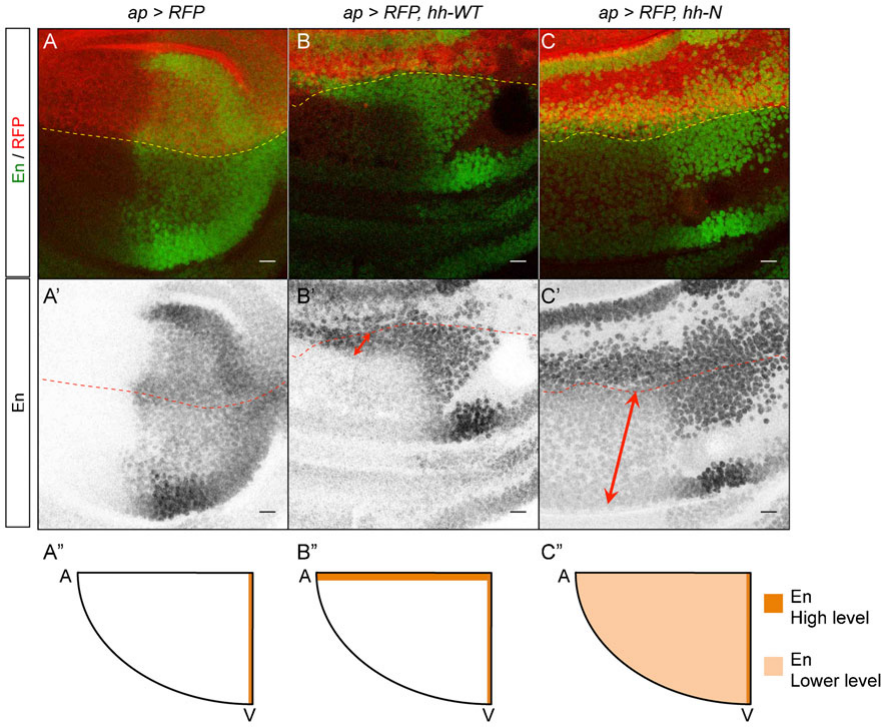
Hh-N influences En patterning at a long-range in the wing *Drosophila* disc. (**A–C′**) Confocal sections presenting En and RFP expression in wing imaginal discs. The expression domain is located above the dashed lines. (A,A′) Control discs. Hh induces En in a 2-cell stripe abutting the A/P border. (B,B′) *ap-Gal4, UAS-RFP, UAS-hh-WT* discs. Ectopic En is detected at a 4-cell range (red arrow). In the rest of the quadrant, En is not detected. (C,C′) *ap-Gal4, UAS-RFP, UAS-hh-N* discs. Ectopic En is detected throughout the anteroventral quadrant (red arrow). (**A″–C″**) Schematics representing anteroventral quadrants of the above genotypes. Ectopic En is in orange. Scale bars: 10 µm.

**Fig. 7.**
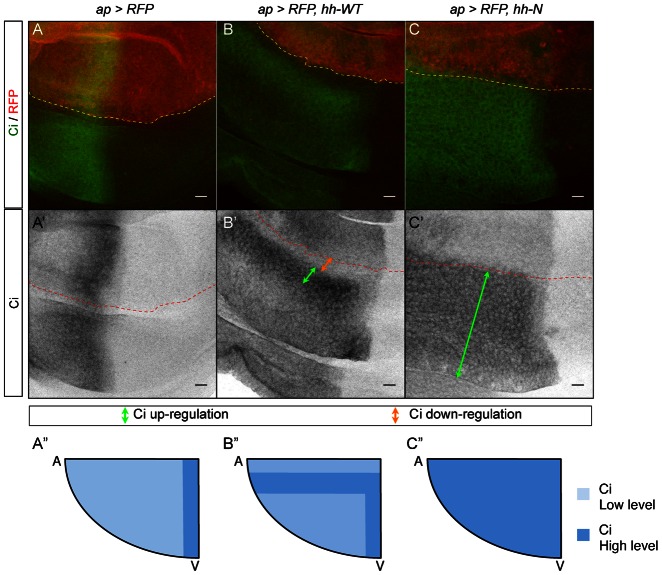
Hh-N influences Ci patterning at a long-range in the wing *Drosophila* disc. (**A–C′**) Ci and RFP expression in wing imaginal discs. The expression domain is located above the dashed lines. (A,A′) Control discs. Hh induces Ci in a 15-cell stripe abutting the A/P border. (B,B′) *ap-Gal4, UAS-RFP, UAS-hh-WT* discs. Ci expression is weak at a 4-cell range where En levels are high, upregulated in the following 10 rows where En is not detected (orange and green arrows respectively). In the rest of the quadrant, Ci level is basal. The endogenous Ci stripe is visible. (C,C′) *ap-Gal4, UAS-RFP, UAS-hh-N* discs. Ci expression is upregulated throughout the quadrant (green arrow), and the endogenous stripe is no more visible. (**A″–C″**) Schematics representing anteroventral quadrants of the above genotypes. Ectopic Ci is in blue. Scale bars: 10 µm.

We therefore decided to analyze the response of the target that requires the highest Hh activity, Engrailed (Blair, 1992). En was also confined to Hh-N expressing clones (Dawber et al., 2005; Callejo et al., 2006; Gallet et al., 2006). In *ap-Gal4, UAS-RFP, UAS-hh-WT* discs, ectopic En is detected in a stripe of 4 cells along the dorsoventral border (Fig. 6B–B″). In *ap-Gal4, UAS-RFP, UAS-hh-N* discs, ectopic En is detected throughout the anteroventral quadrant of the wing pouch (Fig. 6C–C″), albeit at a weaker level compared to Hh-WT discs. Another target of Hh is *cubitus interruptus* (*ci*): *ci* marks the anterior cells, and is upregulated by Hh. Ci is considered a low-threshold target (Dawber et al., 2005). Interestingly, Ci expression is inversely correlated with En expression: The stripe of 4 En cells induced by Hh-WT expresses minimal Ci levels, followed by an area of strong Ci staining that is about 10-cell wide (Fig. 7B–B″). This weaker Ci expression may be due to En-mediated repression. Conversely, Hh-N induces Ci upregulation throughout the anteroventral quadrant (Fig. 7C–C″). Thus the activity plateau generated by Hh-N is strong enough to modify En and Ci patterns, indicating that the longer range of Hh-N does not form at the expense of the activity of the molecule.

**Fig. 8.**
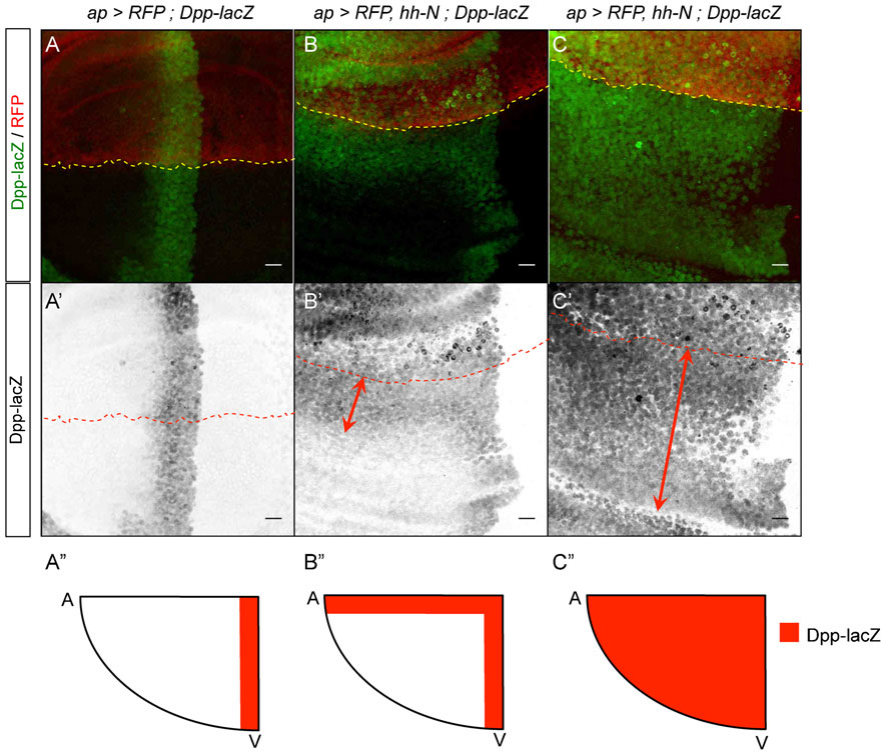
Hh-N induces Dpp-lacZ at a long-range in the *Drosophila* wing disc. (**A–C′**) Dpp-lacZ and RFP expression in wing imaginal discs. The expression domain is located above the dashed lines. (A,A′) Control discs. Hh induces Dpp-lacZ in a 15-cell stripe abutting the A/P border. (B,B′) *ap-Gal4, UAS-RFP, UAS-hh-WT* discs. Ectopic Dpp-lacZ is detected at a 15-cell range (arrows). (C,C′) *ap-Gal4, UAS-RFP, UAS-hh-N* discs. Dpp-lacZ is detected throughout the anteroventral quadrant (arrows). (**A″–C″**) Schematics representing anteroventral quadrants of the above genotypes. Ectopic Dpp-lacZ is in red. Scale bars: 10 µm.

Last, we checked whether Hh-N can induce the low-threshold target *dpp* over a greater range than Hh-WT by analyzing the expression of a *dpp-lacZ* reporter construct (Blackman et al., 1991). Indeed, there is a clear disagreement on whether Hh-N induces dpp-lacZ over a greater range (Callejo et al., 2006) or a reduced range (Gallet et al., 2006) compared with Hh-WT. Our data indicate that whereas Hh-WT induces dpp-lacZ expression in a stripe of about 15 cells along the dorsoventral border, Hh-N induces dpp-lacZ throughout the anterioventral quadrant of the wing pouch (Fig. 8A–C″). As controls, we verified that membrane-anchored Hh induces its targets in a cell-autonomous manner (supplementary material Fig. S4). All the Hh targets we analyzed indicate that cholesterol prevents the formation of a high Hh activity plateau that would cover the full wing pouch. Cholesterol addition is therefore crucial to Hh gradient formation.

## Discussion

### Cholesterol-free Hh acts at long range in both the embryo and the wing imaginal disc

Our data show that cholesterol-free Hh signals at long range. In the embryo, cholesterol-free Hh diffuses and influences patterning at least ten times further than Hh-WT. This clearly contrasts with the generally admitted view that the cholesterol is necessary to send Hh away in the *Drosophila* embryo (Gallet et al., 2003; Gallet et al., 2006) and agrees with pioneer data (Burke et al., 1999). Results showing that cholesterol is necessary to send Hh away may be explained by the fact that Hh does not induce but maintains cell identity in the embryo (Vincent et al., 2008). In experiments performed in Hh null background, target cell identity may have been lost with any delay in Hh-N production, explaining why in these experiments Hh-N would not even act on the very first neighboring cell. Still the novelty of our results resides in the detection of a range that has not been appreciated before: until now, Hh variants were expressed in a striped-pattern and Hh activity was monitored along a maximum range of about 5 cells within each segment (Burke et al., 1999; Gallet et al., 2003; Gallet et al., 2006). Here we show that Hh-N travels at least 25 cells away from its source of secretion and demonstrate for the first time a long-range activity for Hh-N in the *Drosophila* embryo.

In the wing imaginal disc, our data show that cholesterol-free Hh activates at a long range the low-threshold targets such as Dpp, which confirms previous data (Callejo et al., 2006), but also the high-threshold targets such as Ptc and En, which has never been shown before. It has been proposed that the long-range activation of Dpp by Hh-N initially observed by Burke and colleagues would result from ectopic expression of Hh-N in the cells of the peripodial membrane (Gallet et al., 2006). The peripodial cells would secrete Hh-N in the disc lumen, where it would diffuse in a Ptc-independent manner (Callejo et al., 2006). This argument cannot apply against our data: Ap, that drives the Gal4, is the dorsal determinant and is never expressed in the peripodial cells, that are of ventral origin. Thus, Hh-N produced by the dorsal cells of the disc proper is able to travel freely throughout the Ptc expressing epithelium.

### Cholesterol-free Hh can travel through a Ptc expressing territory both in the embryo and the wing imaginal disc

This movement through a Ptc expressing territory in both the embryo and the wing imaginal disc is unexpected. Indeed, Hh-WT moves freely through Ptc minus clones in the wing imaginal disc, indicating that Ptc sequesters Hh-WT (Chen and Struhl, 1996). As both Hh-WT and Hh-N activate signaling, it is assume that both contact Ptc in order to activate the pathway. The movement of Hh-N through a Ptc expressing tissue suggests that Hh and Ptc may undergo several types of interactions: First, a cholesterol-independent interaction would promote signaling. Second, a cholesterol-dependent interaction would promote tethering. Such cholesterol-mediated retention of Hh provides an attractive hypothesis to explain how cholesterol shapes the Hh morphogen gradient.

### Cholesterol binding is required for gradient formation

Still, the most striking result of this analysis is that cholesterol-free Hh leads to the formation of a high Hh activity plateau that extends through the wing pouch. Previous studies concluded that both Hh-N and Hh-WT could establish a gradient and that the function of cholesterol modification is to tune the slope of the gradient (Dawber et al., 2005; Callejo et al., 2006; Gallet et al., 2006; Su et al., 2007). In contrast, our data suggest that cholesterol is not important to refine the gradient as previously believed, but rather is crucial to generate the gradient.

### Robustness as a possible pitfall for morphogen analysis

The vertebrate field provides us with an attractive hypothesis to explain the discrepancy observed in the range of action of Hh-N: Elegant studies about the Sonic Hh (SHh) gradient during the patterning of the neural tube have shown that SHh concentration at a given time is not sufficient to provide spatial information: Aberrant variations in SHh signalling can be ignored, and the memory of the system prevails through a transcription factor feedback loop, a property called hysteresis (Balaskas et al., 2012). The drawback of this robustness is that an experimentally triggered variation in signalling may not give the same result as the same variation performed at steady state. The prediction is that if hysteresis is involved in the fly system, overexpression clones will show different results compared with a steady state overexpression. Several lines of evidence suggest that hysteresis plays an important role in *Drosophila*. First, in the embryo, we have previously shown that Hh does not induce, but maintains groove identity, indicating that memory is crucial to embryonic development (Vincent et al., 2008). Second, the correspondence that we observe between En and Ci expression in the wing imaginal disc indicates that here also a transcription factor loop is at work downstream of Hh signaling. Altogether, steady state analysis appears to be a more appropriate tool than clonal analysis in order to avoid caveats linked to hysteresis.

### Compatibility with the cytoneme model

Cholesterol covalent binding may guide Hh through a specific path to generate an activity gradient (Kornberg, 2011). In this view, cholesterol would function as a barcode in secreting cells to route Hh from the apical membrane to the basal side where cytonemes are produced (Bilioni et al., 2013). In contrast, Hh-N would fail to be targeted basally and would accumulate at the apical surface to be eventually released when the accumulation is too important. This byproduct of Hh synthesis was predicted to generate weakened signaling (Kornberg, 2011). Conversely, our data indicate that Hh-N induces robust levels of high-threshold targets at long distance, arguing against an accidental release. On the other hand, our data may provide a testable hypothesis in order to assess the relevance of cytonemes in Hh gradient formation: As Ptc appears to be specifically required to sequester the cholesterol-bound form, the mechanism that distributes Hh as a gradient should enable Ptc tethering activity: If cytonemes are implicated in Hh movement, they should allow the traveling of Hh through Ptc minus clones and a shift in the position of the gradient. In order to cross Ptc minus clones, cytonemes should either expand or carry a higher number of Hh molecules and resume their wild-type behavior once wild-type tissue is reached. Whereas targeting Hh to cytonemes with cholesterol is an interesting possibility, further experiments need to be performed in order to favor this hypothesis.

Altogether, our data demonstrate unambiguously that Hh without cholesterol diffuses further than Hh-WT in both the embryonic epidermis and the wing imaginal disc. In the embryo, cholesterol binding ensures short-range signaling and in the wing imaginal disc it allows gradient formation. This opens the possibility that a morphogen gradient may not form by the active transfer of a poorly diffusible ligand, but could be generated from the restriction of a highly diffusible ligand.

## Supplementary Material

Supplementary Material

## References

[b1] Alexandre C. Lecourtois M. Vincent J. (1999). Wingless and Hedgehog pattern Drosophila denticle belts by regulating the production of short-range signals. Development 126, 5689–5698. 10572045 10.1242/dev.126.24.5689

[b2] Baker N. E. (1987). Molecular cloning of sequences from wingless, a segment polarity gene in Drosophila: the spatial distribution of a transcript in embryos. EMBO J. 6, 1765–1773. 16453776 10.1002/j.1460-2075.1987.tb02429.x PMC553553

[b3] Balaskas N. Ribeiro A. Panovska J. Dessaud E. Sasai N. Page K. M. Briscoe J. Ribes V. (2012). Gene regulatory logic for reading the Sonic Hedgehog signaling gradient in the vertebrate neural tube. Cell 148, 273–284 10.1016/j.cell.2011.10.047 22265416 PMC3267043

[b4] Bilioni A. Sánchez-Hernández D. Callejo A. Gradilla A. C. Ibáñez C. Mollica E. Carmen Rodríguez-Navas M. Simon E. Guerrero I. (2013). Balancing Hedgehog, a retention and release equilibrium given by Dally, Ihog, Boi and shifted/DmWif. Dev. Biol. 376, 198–212 10.1016/j.ydbio.2012.12.013 23276604

[b5] Blackman R. K. Sanicola M. Raftery L. A. Gillevet T. Gelbart W. M. (1991). An extensive 3′ cis-regulatory region directs the imaginal disk expression of decapentaplegic, a member of the TGF-beta family in Drosophila. Development 111, 657–666. 1908769 10.1242/dev.111.3.657

[b6] Blair S. S. (1992). Engrailed expression in the anterior lineage compartment of the developing wing blade of Drosophila. Development 115, 21–33. 1353439 10.1242/dev.115.1.21

[b7] Brand A. H. Manoukian A. S. Perrimon N. (1994). Ectopic expression in Drosophila. Methods Cell Biol. 44, 635–654 10.1016/S0091-679X(08)60936-X 7707973

[b9] Briscoe J. Chen Y. Jessell T. M. Struhl G. (2001). A hedgehog-insensitive form of patched provides evidence for direct long-range morphogen activity of sonic hedgehog in the neural tube. Mol. Cell 7, 1279–1291 10.1016/S1097-2765(01)00271-4 11430830

[b10] Bürglin T. R. (2008). The Hedgehog protein family. Genome Biol. 9, 241 10.1186/gb-2008-9-11-241 19040769 PMC2614485

[b11] Burke R. Nellen D. Bellotto M. Hafen E. Senti K. A. Dickson B. J. Basler K. (1999). Dispatched, a novel sterol-sensing domain protein dedicated to the release of cholesterol-modified hedgehog from signaling cells. Cell 99, 803–815 10.1016/S0092-8674(00)81677-3 10619433

[b12] Calleja M. Moreno E. Pelaz S. Morata G. (1996). Visualization of gene expression in living adult Drosophila. Science 274, 252–255 10.1126/science.274.5285.252 8824191

[b13] Callejo A. Torroja C. Quijada L. Guerrero I. (2006). Hedgehog lipid modifications are required for Hedgehog stabilization in the extracellular matrix. Development 133, 471–483 10.1242/dev.02217 16396909

[b14] Chamoun Z. Mann R. K. Nellen D. von Kessler D. P. Bellotto M. Beachy P. A. Basler K. (2001). Skinny hedgehog, an acyltransferase required for palmitoylation and activity of the hedgehog signal. Science 293, 2080–2084 10.1126/science.1064437 11486055

[b15] Chen Y. Struhl G. (1996). Dual roles for patched in sequestering and transducing Hedgehog. Cell 87, 553–563 10.1016/S0092-8674(00)81374-4 8898207

[b16] Dawber R. J. Hebbes S. Herpers B. Docquier F. van den Heuvel M. (2005). Differential range and activity of various forms of the Hedgehog protein. BMC Dev. Biol. 5, 21 10.1186/1471-213X-5-21 16197551 PMC1266354

[b17] Dourlen P. Bertin B. Chatelain G. Robin M. Napoletano F. Roux M. J. Mollereau B. (2012). Drosophila fatty acid transport protein regulates rhodopsin-1 metabolism and is required for photoreceptor neuron survival. PLoS Genet. 8, e1002833 10.1371/journal.pgen.1002833 22844251 PMC3405995

[b19] Eugster C. Panáková D. Mahmoud A. Eaton S. (2007). Lipoprotein-heparan sulfate interactions in the Hh pathway. Dev. Cell 13, 57–71 10.1016/j.devcel.2007.04.019 17609110

[b20] Gallet A. Rodriguez R. Ruel L. Therond P. P. (2003). Cholesterol modification of hedgehog is required for trafficking and movement, revealing an asymmetric cellular response to hedgehog. Dev. Cell 4, 191–204 10.1016/S1534-5807(03)00031-5 12586063

[b21] Gallet A. Ruel L. Staccini-Lavenant L. Thérond P. P. (2006). Cholesterol modification is necessary for controlled planar long-range activity of Hedgehog in Drosophila epithelia. Development 133, 407–418 10.1242/dev.02212 16396912

[b22] Glise B. Miller C. A. Crozatier M. Halbisen M. A. Wise S. Olson D. J. Vincent A. Blair S. S. (2005). Shifted, the Drosophila ortholog of Wnt inhibitory factor-1, controls the distribution and movement of Hedgehog. Dev. Cell 8, 255–266 10.1016/j.devcel.2005.01.003 15691766

[b23] Greco V. Hannus M. Eaton S. (2001). Argosomes: a potential vehicle for the spread of morphogens through epithelia. Cell 106, 633–645 10.1016/S0092-8674(01)00484-6 11551510

[b24] Guerrero I. Chiang C. (2007). A conserved mechanism of Hedgehog gradient formation by lipid modifications. Trends Cell Biol. 17, 1–5 10.1016/j.tcb.2006.11.002 17126548

[b25] Ingham P. W. McMahon A. P. (2001). Hedgehog signaling in animal development: paradigms and principles. Genes Dev. 15, 3059–3087 10.1101/gad.938601 11731473

[b26] Ingham P. W. Nakano Y. Seger C. (2011). Mechanisms and functions of Hedgehog signalling across the metazoa. Nat. Rev. Genet. 12, 393–406 10.1038/nrg2984 21502959

[b27] Jessell T. M. (2000). Neuronal specification in the spinal cord: inductive signals and transcriptional codes. Nat. Rev. Genet. 1, 20–29 10.1038/35049541 11262869

[b28] Kornberg T. B. (2011). Barcoding Hedgehog for intracellular transport. Sci. Signal. 4, pe44 10.1126/scisignal.2002447 22114141 PMC4337800

[b29] Kornberg T. B. Guha A. (2007). Understanding morphogen gradients: a problem of dispersion and containment. Curr. Opin. Genet. Dev. 17, 264–271 10.1016/j.gde.2007.05.010 17643982 PMC1993832

[b30] Kornberg T. Sidén I. O'Farrell P. Simon M. (1985). The engrailed locus of Drosophila: in situ localization of transcripts reveals compartment-specific expression. Cell 40, 45–53 10.1016/0092-8674(85)90307-1 3917856

[b31] Mann R. K. Beachy P. A. (2004). Novel lipid modifications of secreted protein signals. Annu. Rev. Biochem. 73, 891–923 10.1146/annurev.biochem.73.011303.073933 15189162

[b32] McMahon A. P. Ingham P. W. Tabin C. J. (2003). Developmental roles and clinical significance of hedgehog signaling. Curr. Top. Dev. Biol. 53, 1–114 10.1016/S0070-2153(03)53002-2 12509125

[b33] Micchelli C. A. The I. Selva E. Mogila V. Perrimon N. (2002). Rasp, a putative transmembrane acyltransferase, is required for Hedgehog signaling. Development 129, 843–851. 11861468 10.1242/dev.129.4.843

[b35] Nüsslein-Volhard C. Wieschaus E. (1980). Mutations affecting segment number and polarity in Drosophila. Nature 287, 795–801 10.1038/287795a0 6776413

[b36] Panáková D. Sprong H. Marois E. Thiele C. Eaton S. (2005). Lipoprotein particles are required for Hedgehog and Wingless signalling. Nature 435, 58–65 10.1038/nature03504 15875013

[b37] Porter J. A. Young K. E. Beachy P. A. (1996a). Cholesterol modification of hedgehog signaling proteins in animal development. Science 274, 255–259 10.1126/science.274.5285.255 8824192

[b38] Porter J. A. Ekker S. C. Park W. J. von Kessler D. P. Young K. E. Chen C. H. Ma Y. Woods A. S. Cotter R. J. Koonin E. V. (1996b). Hedgehog patterning activity: role of a lipophilic modification mediated by the carboxy-terminal autoprocessing domain. Cell 86, 21–34 10.1016/S0092-8674(00)80074-4 8689684

[b39] Ramírez-Weber F. A. Kornberg T. B. (1999). Cytonemes: cellular processes that project to the principal signaling center in Drosophila imaginal discs. Cell 97, 599–607 10.1016/S0092-8674(00)80771-0 10367889

[b40] Ranieri N. Ruel L. Gallet A. Raisin S. Thérond P. P. (2012). Distinct phosphorylations on kinesin costal-2 mediate differential hedgehog signaling strength. Dev. Cell 22, 279–294 10.1016/j.devcel.2011.12.002 22306085

[b41] Riddle R. D. Johnson R. L. Laufer E. Tabin C. (1993). Sonic hedgehog mediates the polarizing activity of the ZPA. Cell 75, 1401–1416 10.1016/0092-8674(93)90626-2 8269518

[b42] Rojas-Ríos P. Guerrero I. González-Reyes A. (2012). Cytoneme-mediated delivery of hedgehog regulates the expression of bone morphogenetic proteins to maintain germline stem cells in Drosophila. PLoS Biol. 10, e1001298 10.1371/journal.pbio.1001298 22509132 PMC3317903

[b43] Roy S. Hsiung F. Kornberg T. B. (2011). Specificity of Drosophila cytonemes for distinct signaling pathways. Science 332, 354–358 10.1126/science.1198949 21493861 PMC3109072

[b44] Strigini M. Cohen S. M. (1997). A Hedgehog activity gradient contributes to AP axial patterning of the Drosophila wing. Development 124, 4697–4705. 9409685 10.1242/dev.124.22.4697

[b45] Su V. F. Jones K. A. Brodsky M. The I. (2007). Quantitative analysis of Hedgehog gradient formation using an inducible expression system. BMC Dev. Biol. 7, 43 10.1186/1471-213X-7-43 17484784 PMC1885436

[b46] Tabata T. Kornberg T. B. (1994). Hedgehog is a signaling protein with a key role in patterning Drosophila imaginal discs. Cell 76, 89–102 10.1016/0092-8674(94)90175-9 8287482

[b47] Taylor A. M. Nakano Y. Mohler J. Ingham P. W. (1993). Contrasting distributions of patched and hedgehog proteins in the Drosophila embryo. Mech. Dev. 42, 89–96 10.1016/0925-4773(93)90101-3 8369225

[b48] Vincent S. Perrimon N. Axelrod J. D. (2008). Hedgehog and Wingless stabilize but do not induce cell fate during Drosophila dorsal embryonic epidermal patterning. Development 135, 2767–2775 10.1242/dev.017814 18614578 PMC2585068

[b49] Wendler F. Franch-Marro X. Vincent J. P. (2006). How does cholesterol affect the way Hedgehog works? Development 133, 3055–3061 10.1242/dev.02472 16873581

