# Supplementary material for: Absolute requirement of cholesterol binding for Hedgehog gradient formation in Drosophila
Source: Biol Open. 2013 May 9;2(6):596–604. doi: 10.1242/bio.20134952 (PMC3683162; doi:10.1242/bio.20134952)
Supplement: Supplementary Material [file supp_2_6_596__index.html]

Absolute requirement of cholesterol binding for Hedgehog gradient formation in Drosophila — Absolute requirement of cholesterol binding for Hedgehog gradient formation in Drosophila — Supplementary Material 

# Absolute requirement of cholesterol binding for Hedgehog gradient formation in *Drosophila*

## bio.20134952 Supplementary Material

**Files in this Data Supplement:**

- Supplementary Material - Antoine Ducuing et al. doi: 10.1242/bio.20134952
