# Supplementary material for: Absolute requirement of cholesterol binding for Hedgehog gradient formation in Drosophila
Source: Biol Open. 2013 May 9;2(6):596–604. doi: 10.1242/bio.20134952 (PMC3683162; doi:10.1242/bio.20134952)
Supplement: Supplementary Material [file supp_bio.20134952_bio.20134952-s1.pdf]

Biology Open

Supplementary Material

Antoine Ducuing et al. **doi: 10.1242/bio.20134952**

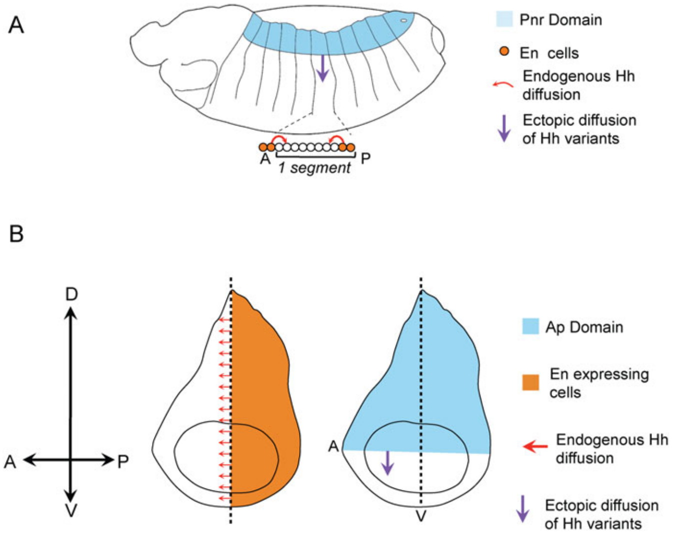

Fig. S1. Cholesterol-free Hh activates Hh targets at a long-range in the *Drosophila* embryo. (**A**) Experimental strategy in the *Drosophila* embryo. Hh is secreted by En cells and diffuses in the *Ci* domain along the anteroposterior axis. In order to monitor Hh diffusion across a higher number of cells, *UAS-hh* variants were expressed in the dorsal epidermis with the *pnr-Gal4* driver and the diffusion was monitored in the lateral and the ventral epidermis (purple arrow). (**B**) Experimental strategy in the *Drosophila* wing imaginal disc. Hh is secreted by En cells (orange) in the posterior compartment and diffuses in the anterior compartment. In order to be independent of endogenous Hh activity and to avoid peripodial membrane expression, *UAS-hh* variants were expressed in dorsal cells (blue) with the *ap-Gal4* driver and the diffusion was monitored along the anteroventral quadrant (purple arrow).

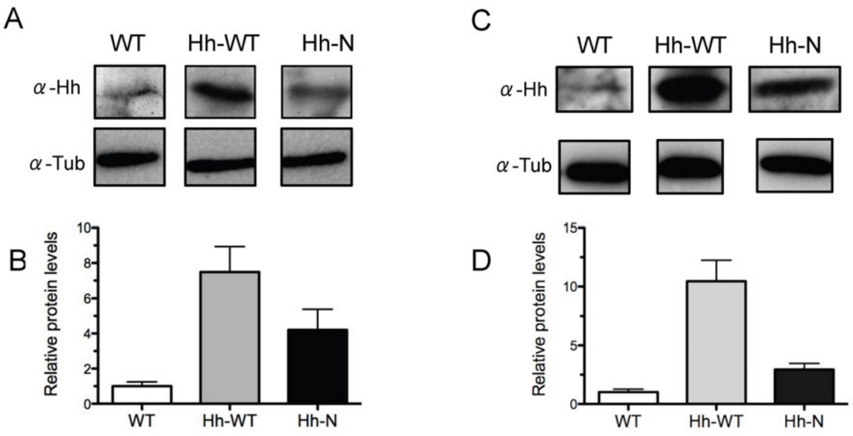

Fig. S2. Quantification of Hh-WT and Hh-N protein levels. (**A**) Western blot analysis of *WT*, *pnr-Gal4, UAS-RFP, UAS-hh-WT* and *pnr-Gal4, UAS-RFP, UAS-hh-N* embryos. Tubulin was used as a loading control. (**B**) Quantification of protein levels. In this system, Hh-WT is expressed at higher level than Hh-N. (**C**) Western blot analysis of *WT*, a*p-Gal4, UAS-RFP, UAS-hh-WT* and *ap-Gal4, UAS-RFP, UAS-hh-N* wing imaginal discs. Tubulin was used as a loading control. (**D**) Quantification of protein levels. In this system, Hh-WT is expressed at higher level than Hh-N.

Biology Open

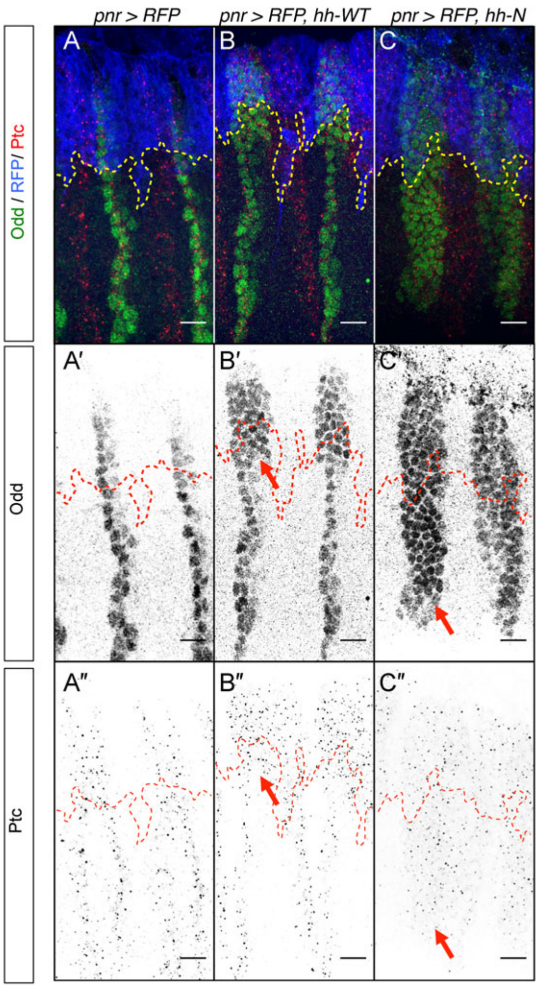

Fig. S3. Expended version of Fig. 2A–C′ showing individual images of Ptc. (**A–A″**) *pnr-Gal4, UAS-RFP*, (**B–B″**) *pnr-Gal4, UAS-RFP, UAS-hh-WT* (**C–C″**) and *pnr-Gal4, UAS-RFP, UAS-hh-N* stage 13 embryos stained for Odd and Ptc. RFP is in blue. Note that in embryos overexpressing Hh-WT Ptc and Odd are detected at a 4-cell range inside the lateral epidermis (arrows). In embryos overexpressing Hh-N, the ectopic Ptc and Odd are detected throughout the lateral epidermis (arrows). Scale bars: 10 µm.

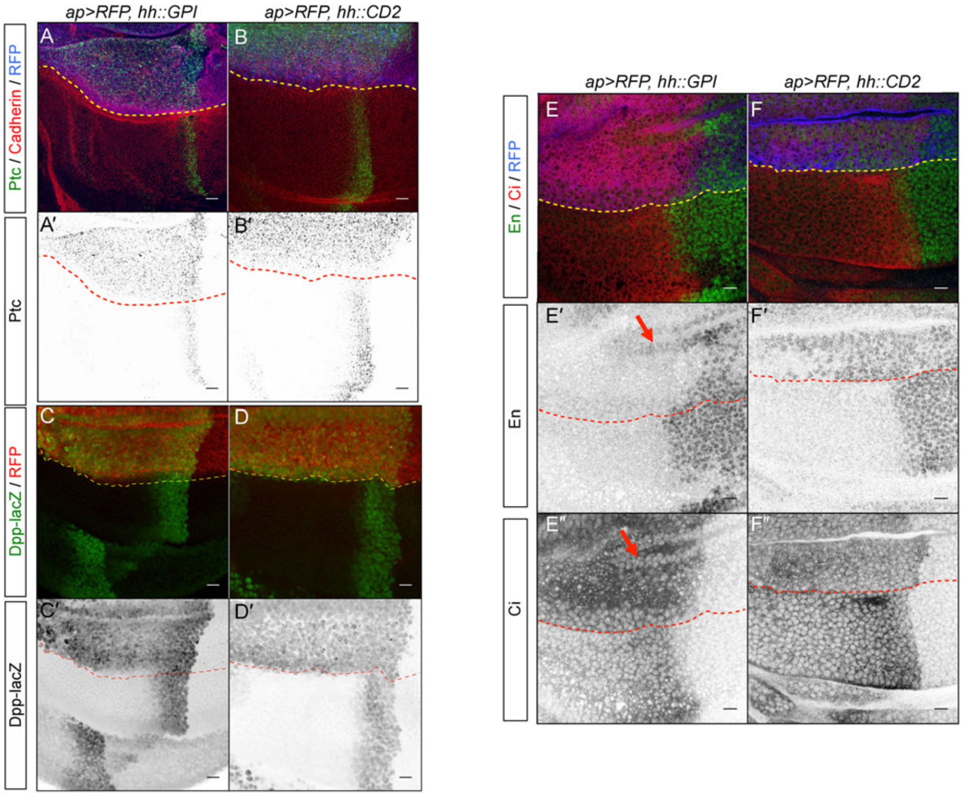

Fig. S4. Hh::GPI and Hh::CD2 activate Ptc, En, Ci and Dpp-lacZ expression in a cellautonomous manner. (**A–B′**) Ptc, Cadherin and RFP, (**C–D′**) Dpp-lacZ and RFP and (**E–F″**) En, Ci and RFP expression in wing imaginal discs. The Apterous domain is located above the dashed lines. Hh::GPI produced in the dorsal domain induces Ptc, dpp-lacZ and Ci upregulation in a cellautonomous manner only. In some cells within the expression domain, (arrows) En is expressed and Ci expression is decreased. Hh::CD2 produced in the dorsal domain induces Ptc, dpp-lacZ and En ectopic expression in a cell-autonomous manner only. Ci is also slightly upregulated in the expression domain only. Scale bars: 10 µm.
